# Preventive Effects of *Chrysanthemum coronarium L.* Extract on Bone Metabolism *In Vitro* and *In Vivo*

**DOI:** 10.1155/2020/6975646

**Published:** 2020-11-18

**Authors:** So Ah Kim, Ae Sin Lee, Haeng Jeon Hur, Sang Hee Lee, Mi Jeong Sung

**Affiliations:** ^1^Research Group of Natural Materials and Metabolism, Food Functionality Research, Korea Food Research Institute, Jeonju, Jeollabuk-Do, Republic of Korea; ^2^Department of Food Science and Technology, Chonbuk National University, Jeonju, Jeollabuk-Do, Republic of Korea

## Abstract

Osteoporosis is characterized by decreased bone mass and bone microarchitectural failure, leading to an enhanced risk of bone fractures. *Chrysanthemum coronarium L*. (CC) is a natural plant with powerful antioxidant activity. This study investigated the antiosteoporotic effects of CC extracts in *in vitro* cell cultures and *in vivo* bone loss animal models. CC stimulated osteoblast differentiation and mineralized bone formation by osteoblasts by increasing the expression of bone formation markers (alkaline phosphatase, osteoprotegerin, and osteoprotegerin/receptor activator nuclear factor-*κ*B ligand ratio) in the murine preosteoblastic cell line MC3T3-E1. Additionally, CC was found to inhibit osteoclast differentiation by downregulating bone resorption markers (tartrate-resistant acid phosphatase, cathepsin K, dendritic cell-specific transmembrane protein, and calcitonin receptor) in the murine macrophage-like cell line RAW264.7. CC prevented ovariectomy-induced bone loss, preserved trabecular microarchitecture, and improved serum bone turnover markers in an osteoporotic mouse model. These findings suggest that CC extract may be considered as a natural therapeutic or preventive agent for osteoporotic bone loss.

## 1. Introduction

Bone is an active living organ that is constantly renewed, with maintained homeostasis, which is a closely regulated physiological process characterized by osteogenesis of osteoblasts and osteoclastogenesis of osteoclasts [1–3]. Osteoblasts derived from mesenchymal stem cells are responsible for bone formation by the synthesis and mineralization of the bone matrix [4]. Osteoclasts are bone-resorbing multinucleated cells derived from monocytes/macrophages [5]. However, an imbalanced regulation of bone resorption and bone formation may occur, leading to abnormal bone remodeling and osteoporosis [3].

Osteoporosis is characterized by reduced bone mass and impaired bone quality, leading to an enhanced risk of bone fractures. It mainly affects elderly people and/or postmenopausal women [6, 7]. Currently, numerous pharmacological drugs, including bisphosphonates and parathyroid hormones, are available as potential therapeutics for osteoporosis [8, 9].

Various herbal medicines from natural products and traditional foods are known to improve bone density and reduce the risk of bone fracture, thereby facilitating their use in the treatment of osteoporosis [10–12]. *Glebionis coronaria* (*Chrysanthemum coronarium* L., crown daisy, CC), known as “ssukgat” in Korea, is commonly distributed in the Mediterranean region, Western Africa, and Asia. CC is a leafy vegetable with a potent antioxidant capacity [13–15]. Howaida et al. reported that CC possesses various beneficial effects, including antifungal, antihypercholesterolemic, and antihyperglycemic effects [16]. In addition, it was recently reported that fermented CC has antiaging effects *in vitro* [17]. However, the effects of CC on bone metabolism are still unclear.

In our research, we aimed to identify the effect of CC on osteoclast formation in the murine macrophage-like cell line RAW 264.7 and on osteoblast differentiation in the murine preosteoblastic cell line MC3T3-E1. Furthermore, we identified the preventive and therapeutic effects of CC in a mouse model with ovariectomy- (OVX-) induced bone loss.

## 2. Materials and Methods

### 2.1. Preparation and Analysis of CC

Dried CC was obtained from a local market in Korea and subjected to extraction using 50% ethanol (1 : 10 w/v) at room temperature for 24 h. The extract was filtered using filter paper and lyophilized by freeze-drying at −80°C. Analysis and identification of the major compounds in the CC extract were performed by ultraperformance liquid chromatography-quadrupole time-of-flight mass spectrometry (UPLC-Q-TOF MS; Waters, Milford, MA, USA) with an Acquity UPLC BEH C18 column (100 × 2.1 mm, 1.7 *μ*m; Waters). The extract was injected onto the column equilibrated with water/acetonitrile (99 : 1) containing 0.1% formic acid and eluted with a linear gradient (1–100%) of acetonitrile containing 0.1% formic acid at a flow rate of 0.35 mL/min for 9 min at 40°C column temperature. The eluted compounds were analyzed by Q-TOF MS in the negative electrospray ionization mode. The scan range of TOF MS data was 50–1500 m/*z*, the scan time was 0.2 sec, and the capillary and sampling cone voltages were 2.5 kV and 40 V, respectively. The desolvation flow rate and temperature were 900 L/h and 400°C, respectively, and the source temperature was 100°C. LockSpray with leucine-enkephalin ([*M* + *H*] = 554.2615 Da) was used to ensure the reproducibility and accuracy of all analyses. MS/MS spectra were obtained under collision energy ramp (10–30 eV). Metabolites were identified based on online databases, including the Chemspider database in UNIFI 1.8.2 (Waters) and the METLIN database (http://www.metlin.scripps.edu).

### 2.2. Cell Culture and Differentiation

MC3T3-E1 and RAW 264.7 cells were purchased from the American Type Culture Collection (ATCC, Manassas, VA, USA). The *α*-Minimum essential medium (*α*-MEM), Dulbecco's modified Eagle's medium (DMEM), and fetal bovine serum (FBS) were obtained from Invitrogen Co. (Grand Island, NY, USA). Receptor activator nuclear-factor *κ*B ligand (RANKL) was purchased from R&D Systems (Minneapolis, MN, USA). Ascorbic acid (AA) and *β*-glycerophosphate (*β*-GP) were purchased from Sigma (St. Louis, MO, USA), unless otherwise indicated. MC3T3-E1 cells were cultured in *α*-MEM, whereas RAW 264.7 cells were cultured in DMEM supplemented with 10% FBS. For osteoblast differentiation, MC3T3-E1 cells were stimulated in *a*-MEM supplemented with AA (50 *μ*g/mL) and *β*-GP (10 mM) in the presence or absence of various concentrations of CC extracts. For osteoclast differentiation, RAW 264.7 cells were stimulated in DMEM supplemented with RANKL (50 ng/mL) in the presence or absence of various concentrations of CC extracts. The cells were maintained in a humidified atmosphere of 5% CO_2_ at 37°C. The cell culture medium with/without CC extracts was changed every three days.

### 2.3. Cell Viability

MC3T3-E1 cells (3 × 10^3^ cells/mL in a 96-well plate) and RAW 264.7 cells (1 × 10^4^ cells/mL in a 96-well plate) were seeded and treated with or without various concentrations of CC extracts. Cell viability was analyzed using the 3-(4,5-dimethylthiazol-2-yl)-2,5-diphenyltetrazolium bromide assay (Sigma-Aldrich, St. Louis, MO, USA) according to the supplier's recommendation. The absorbance was measured at 450 nm with a microplate reader (Versamax; Molecular Devices, CA, USA).

### 2.4. Tartrate-Resistant Acid Phosphatase (TRAP) Staining

After induction, RAW 264.7 cells were treated with RANKL and various concentrations of CC extracts. After induction of osteoclast differentiation for 4 d, the cells were stained using the Acid Phosphatase Leukocyte Kit (Sigma-Aldrich). Then, TRAP-positive multinucleated cells (3 nuclei) were considered as osteoclasts and counted.

### 2.5. Alkaline Phosphatase (ALP) Activity and Mineralization Assay

After induction of osteoblast differentiation for 6 d, MC3T3-E1 cells were washed with phosphate-buffered saline (PBS) and lysed using 0.2% Triton-X 100 lysis buffer at 4°C. ALP activity was assessed using the ALP detection kit (Bio-Rad Laboratories Inc., Hercules, CA) according to the manufacturer's protocols. MC3T3-E1 cells were, then, induced for osteoblast differentiation in the presence or absence of different concentrations of CC extracts for three weeks. The cells were fixed in 70% ethanol for 10 min and stained with alizarin red S (Sigma-Aldrich). Calcium deposits in the mineralized cells were observed by using a light microscope (Nikon, Tokyo, Japan).

### 2.6. Quantitative Reverse Transcription-Polymer Chain Reaction (RT-qPCR) Analysis

MC3T3-E1 cells were stimulated with AA and *β*-GP and treated with or without various concentrations of CC extracts for 2 d. RAW 264.7 cells were stimulated with RANKL and treated with or without various concentrations of CC extracts for 2 days. Total cellular RNA was extracted from the cultured cells using the QIAGEN mini kit (Qiagen, Valencia, CA, USA) according to the manufacturer's recommendation and reverse-transcribed to cDNA using the cDNA synthesis kit ReverTra Ace qPCR RT Master Mix (ToYoBo, Osaka, Japan). The reaction was performed using the iTaq universal SYBR Green I supermix (Bio-Rad Laboratories Inc.) according to the manufacturer's instruction. The PCR primer sequences are listed in Table 1. The relative quantification was normalized against the level of glyceraldehyde 3-phosphate dehydrogenase (GAPDH). The relative transcript expression was calculated using the 2^−DCT^ method, and results were expressed as folds of change relative to the expression in the control group [18].

### 2.7. Animals and Ovariectomy

Eight-week-old female ICR mice were provided by Orient-Bio Korea (Seoul, Korea). Mice were randomly divided into the following four groups (*n* = 10): vehicle-treated SHAM (SH), vehicle-treated OVX (OVX), vehicle-treated estrogen (*O* + Es, 0.4 mg/kg in the diet), and CC-treated OVX (*O* + CC, 5 g/kg in the diet) groups [19]. Bilateral ovariectomy was performed in the OVX groups, whereas ovaries were not removed in the sham-operated group. Mice were allowed to recover for one week. The experimental diet used was AIN 93G diet (Dyets Inc., Bethlehem, PA, USA). Mice were allowed free access to a soy-free diet and CC diet for 12 weeks. Thereafter, mice were sacrificed under euthanasia (100 mg/kg ketamine and 5 mg/kg xylazine), and blood samples were collected via heart puncture. All animal procedures were approved by the Institutional Animal Care and Use Committee of the Korea Food Research Institute (KFRI: KFRI-M-17043) and carried out in accordance with relevant guidelines and regulations.

### 2.8. Analysis of Serum Bone Biomarkers

Serum levels of ALP and osteocalcin were determined using commercial enzyme-linked immunosorbent assay (ELISA) kits (Biomedical Technologies, Stoughton, MA, USA). The levels of deoxypyridinoline (DPD) were also assessed with a commercial kit (Kamiya Biochemical Company, Seattle, WA, USA). All the assays were performed according to the manufacturer's recommendations.

### 2.9. Measurement of Bone Mass Density (BMD) and Microcomputed Tomography (*µ*CT) Scanning

BMD of the total femurs was assessed by dual-energy X-ray absorptiometry with a Norland pDEXA Sabre (Norland Medical Systems Inc., Fort Atkinson, WI, USA) equipped with the appropriate software for small animals. The region of interest comprised the distal femur from the proximal growth plate in the distal direction (68 *μ*m/slice). The distal femurs were scanned using a SkyScan 1076 *µ*-CT scanner (Bruker, Karlsruhe, Germany) to determine the bone microarchitecture. The structural changes in bone architecture were measured using the CT Analyzer V 1.11.0.0 (Skyscan, Kontich, Belgium). Bone morphometric parameters were characterized by measuring the bone volume to tissue volume ratio (BV/TV), trabecular thickness (Tb.Th), trabecular number (Tb.N), and trabecular separation (Th.Sp).

### 2.10. Statistical Analysis

*In vitro* data are expressed as means ± standard deviation (SD), whereas *in vivo* data are expressed as means ± standard error mean (SEM). The statistical significance of parameters was assessed by one-way analysis of variance (ANOVA) combined with Tukey's post-hoc test using Graph Pad Prism software version 6.05 (Graph Pad Inc., La Jolla, CA, USA). A *p* value < 0.05 was considered statistically significant.

## 3. Results

### 3.1. UPLC-Q-TOF Mass Analysis

**Figure 1:**
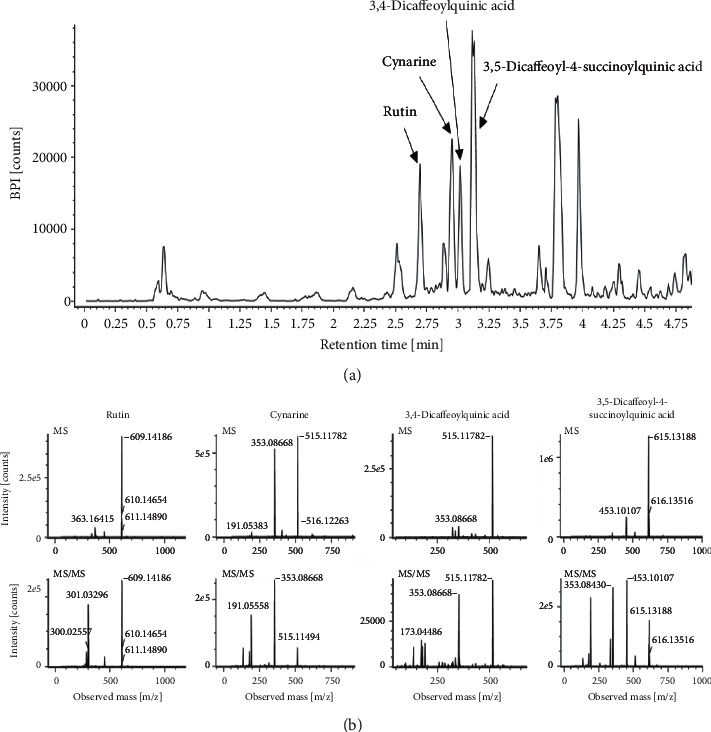
Representative liquid chromatogram of CC (a) and identification of its major compounds (b). The CC extract was analyzed using UPLC-Q-TOF MS, and its major compounds were identified based on the online databases Chemspider (http://www.metlin.scripps.edu) and human metabolome database (http://www.hmdb.ca).

To analyze the compounds present in CC extracts, we used UPLC-Q-TOF mass. The results showed the presence of rutin, cynarine, 3,4-dicaffeoylquinic acid, and 3,5-dicaffeoyl-4-succinoyl quinic acid in CC extracts (Figure 1).

### 3.2. CC Stimulates Osteoblast Differentiation

**Figure 2:**
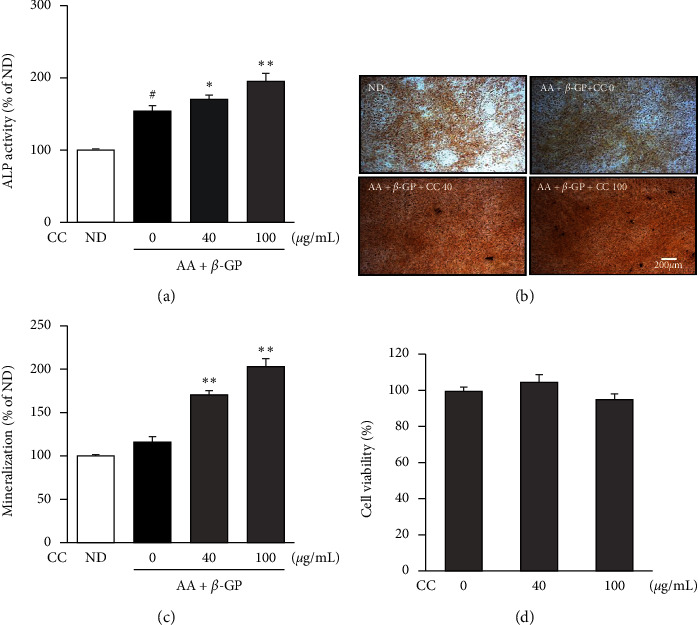
The effect of *Chrysanthemum coronarium* on osteoblast differentiation in the murine preosteoblastic cell line MC3T3-E1. (a) MC3T3-E1 cells were incubated with 50 *μ*g ascorbic acid (AA) and 10 mM *β*-glycerophosphate (*β*-GP) along with (C) coronarium. CC for 4 d alkaline phosphatase (ALP) activity was detected by measuring the protein concentration. (b) MC3T3-E1 cells were incubated with 50 *μ*g AA and 10 mM *β*-GP along with CC for 21 d mineralized nodules were assessed by means of Alizarin red S staining. The mineralized nodules were visualized under a microscope. Scale bar, 200 *μ*m. (c) Alizarin red O optical density was assessed. (d) Cells were treated with different concentrations of CC for 4 d, and the cell viability was measured. ND, nondifferentiated. Data are represented as means ± SD, *n* = 3, ^#^*p* < 0.05 compared with the control group, and ^*∗*^*p* < 0.05 and ^*∗∗*^*p* < 0.01 compared with the AA- and *β*-GP treatment group. CC increases the expression of osteoblast-related genes.

To evaluate the effect of CC on osteoblast differentiation, we measured ALP activity in MC3T3-E1 cells. AA- and *β*-GP-treated cells differentiated into osteoblasts, which stimulated ALP activity. CC was found to increase the AA- and *β*-GP-induced ALP activity in a dose-dependent manner on day 4 (Figure 2(a)). Next, to determine bone mineralization, we performed alizarin red S staining. CC increased mineralization in a concentration-dependent manner after incubation for 21 d (Figures 2(b) and 2(c)). Moreover, CC was not toxic to differentiated osteoblasts (Figure 2(d)). These findings suggest that CC promotes the differentiation and mineralization of osteoblastic cells.

**Figure 3:**
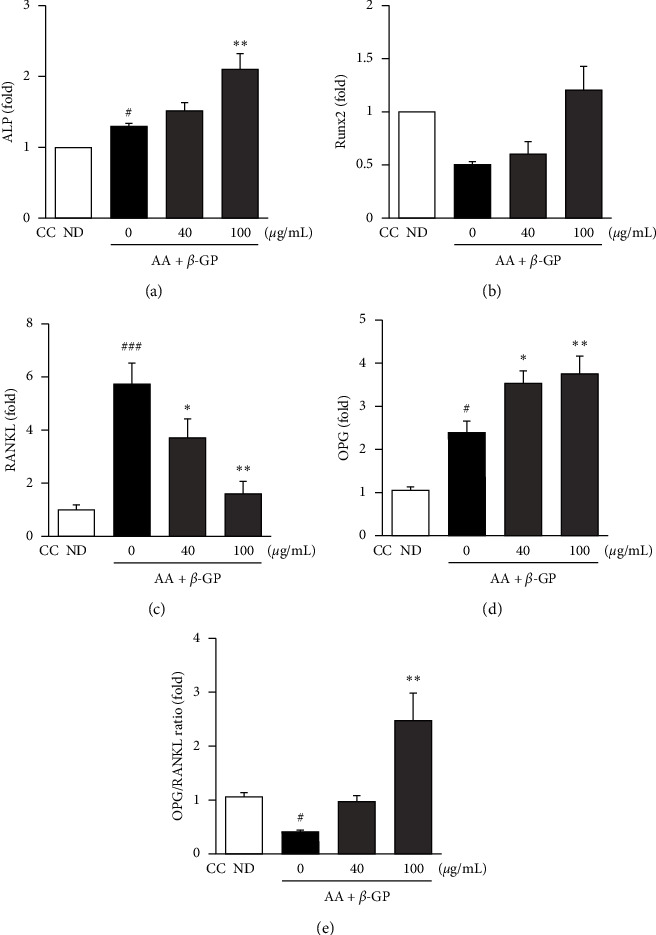
The effect of *Chrysanthemum coronarium* on osteoblastic gene expression in the murine preosteoblastic cell line MC3T3-E1. (A–E) MC3T3-E1 cells were incubated with 50 *μ*g ascorbic acid (AA) and 10 mM *β*-glycerophosphate (*β*-GP) at various concentrations for 2 d The total RNA of the cells was extracted, and the mRNA expression of the (a) alkaline phosphatase (ALP), (b) runt-related transcription factor-2 (Runx2), (c) receptor activator nuclear factor- *κ*B ligand (RANKL), and (d) osteoprotegerin (OPG) genes and the (e) OPG/RANKL ratio was assessed using RT-qPCR. RNA expression levels were normalized to those of glyceraldehyde 3-phosphate dehydrogenase (GAPDH). ND, nondifferentiated. Data are represented as means ± SD, *n* = 3, ^#^*p* < 0.05 and ^###^*p* < 0.001 compared with the control group, and ^*∗*^*p* < 0.05 and ^*∗∗*^*p* < 0.01 compared with the AA and *ß*-GP treatment group.

Osteoblast differentiation is mediated via the expression of specific osteoblastic genes, such as ALP and runt-related transcription factor-2 (Runx2) [20]. To elucidate the effects of CC on cellular osteoblastogenesis, we evaluated the mRNA expression of these genes after two days of differentiation using RT-qPCR. CC markedly increased the mRNA expression levels of ALP in a concentration-dependent manner. However, there was no significant difference in Runx2 gene expression compared to its expression in nondifferentiated cells (Figures 3(a) and 3(b)). These findings suggest that CC promotes the upregulation of ALP mRNA expression. The osteoprotegerin (OPG)/RANKL ratio plays an essential role in regulating bone remodeling. CC treatment significantly increased the mRNA expression levels of OPG in a concentration-dependant manner, whereas it markedly inhibited that of RANKL (Figures 3(c) and 3(d)). In addition, CC markedly increased the OPG/RANKL ratio in MC3T3-E1 cells (Figure 3(e)).

### 3.3. CC Inhibits Osteoclast Differentiation

**Figure 4:**
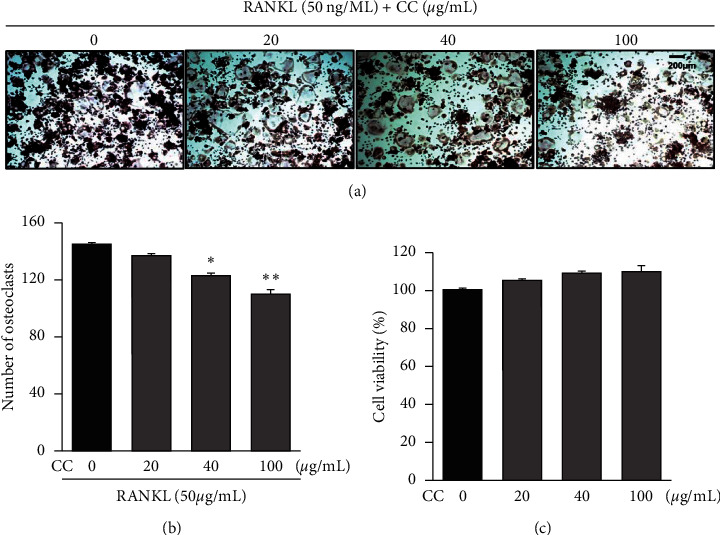
The effect of *Chrysanthemum coronarium* (L) on receptor activator nuclear-factor *κ*B ligand-induced osteoclast differentiation in the murine macrophage-like cell line RAW 264.7. The cells were treated with the indicated concentrations of (C) coronarium (CC), followed by stimulation with receptor activator nuclear-factor *κ*B ligand (RANKL) (50 ng/mL) for 4 d. (a) Tartrate-resistant acid phosphatase (TRAP)-positive cells were visualized under a light microscope. Scale bar, 200 *μ*m. (b) TRAP-positive cells with more than three nuclei were counted. (c) Cell viability in the presence of different concentrations of CC was assessed. Data are represented as means ± SD, *n* = 3, ^*∗*^*p* < 0.05, ^*∗∗*^*p* < 0.01 compared with the RANKL treatment group. CC suppresses the expression of osteoclast-mediated genes.

To evaluate the effects of CC on osteoclastogenesis, we treated RAW 264.7 cells with RANKL at different concentrations of CC. RANKL significantly induced the formation of TRAP-positive osteoclasts, and CC treatment was found to decrease this effect in a dose-dependent manner on day 4 (Figures 4(a) and 4(b)). In addition, CC was not toxic to RAW 264.7 cells on day 4 (Figure 4(c)). These findings suggest that CC extract distinctly inhibits TRAP-positive multinucleated osteoclasts.

**Figure 5:**
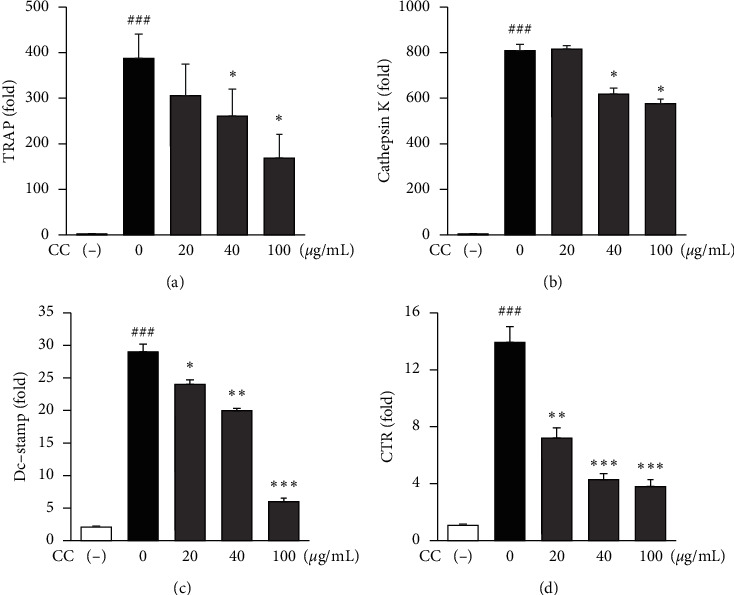
The effect of *Chrysanthemum coronarium* on receptor activator nuclear-factor *κ*B ligand-induced osteoclastic gene expression in the murine macrophage-like cell line RAW 264.7. (A–D) The cells were cultured with receptor activator nuclear-factor *κ*B ligand (RANKL) (50 ng/mL) at various concentrations of CC for 4 d and osteoclast-specific gene expression was determined. (a) Cathepsin K. (b) Tartrate-resistant acid phosphatase (TRAP), (c) dendritic cell-specific transmembrane protein (Dc-Stamp), and (d) calcitonin receptor (CTR) expressions were analyzed using RT-qPCR. RNA expression levels were normalized to those of glyceraldehyde 3-phosphate dehydrogenase (GAPDH). Data represent means ± SD, *n* = 3; ^#^*p* < 0.05 compared with the control group, ^*∗*^*p* < 0.05, ^*∗∗*^*p* < 0.01, and ^*∗∗∗*^*p* < 0.001 compared with the RANKL treatment group.

Osteoclast differentiation is associated with the upregulation of specific genes, including TRAP, cathepsin K, dendritic cell-specific transmembrane protein (Dc-Stamp), and calcitonin receptor (CTR) [21]. To determine the effect of CC on osteoclast differentiation via regulation of osteoclast-associated gene expression, the mRNA expression of these genes was determined by RT-qPCR. The results showed that CC significantly reduced RANKL-induced mRNA levels of these genes in a dose-dependent manner (Figures 5(a)–5(d)). These findings suggest that CC inhibits osteoclast differentiation by suppressing osteoclast-mediated gene expression.

CC prevents the increase in body and fat weights and the decrease in uterine weight in an OVX-induced bone loss mouse model.

The initial body weights and food efficacy rates (FERs) were similar in all groups. OVX mice had a higher final body weight than SHAM mice. However, CC-treated mice exhibited a significantly lower final body weight than OVX mice. These results indicate that CC treatment suppressed OVX-induced body weight increase. OVX mice had a higher fat weight than SHAM mice; however, this higher fat weight was considerably reduced in CC-treated and Es-treated mice. Furthermore, OVX mice had a decreased uterine weight compared with that of SH mice. However, uterine weight was considerably increased in CC-treated mice (Table 2).

### 3.4. CC Inhibits the Bone Loss in a Mouse Model of OVX-Induced Bone Loss

**Figure 6:**
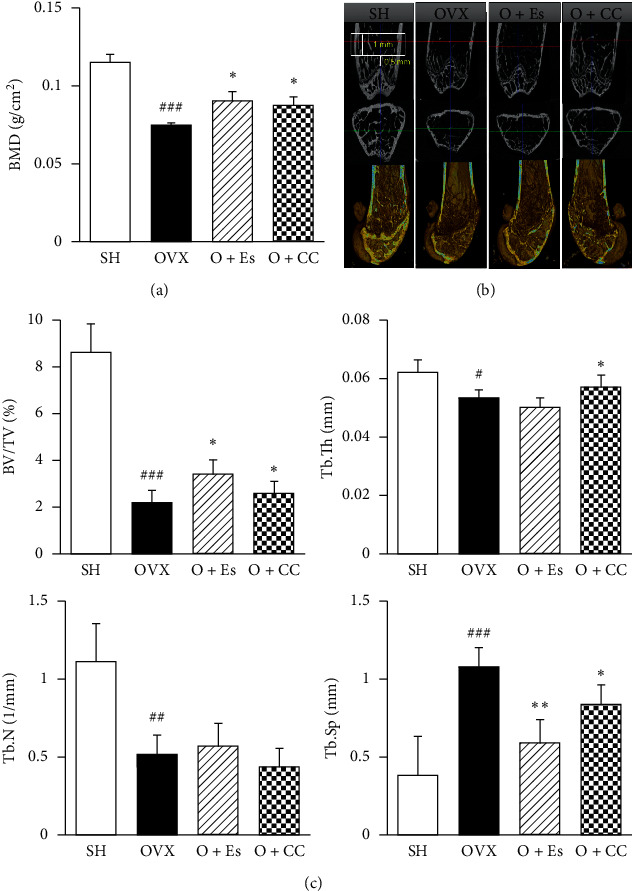
The effect of *Chrysanthemum coronarium* on OVX-induced bone loss in mice. (a) Bone mass density (BMD) of the total femur was measured by dual-energy X-ray absorptiometry. (b) Representative two-dimensional (upper and middle sections) and three-dimensional (lower section) images of the trabecular bone in femurs were analyzed. The yellow box indicates the region of interest. (c) Bone volume to tissue volume (BV/TV) and bone parameters of the femur (trabecular thickness (Tb. Th), trabecular number (Tb. N), and trabecular separation (Tb. Sp)). Data are represented as means ± SEM. SH (sham), sham mice; OVX (ovariectomy), OVX mice; *O* + Es (estrogen), OVX + Es treatment mice; *O* + CC (*Chrysanthemum coronarium*), and OVX + CC treatment mice. Data are represented as means ± SEM, *n* = 8. ^#^*p* < 0.05, ^##^*p* < 0.01, and ^###^*p* < 0.001 compared with SH groups, ^*∗*^*p* < 0.05 and ^*∗∗*^*p* < 0.01 compared with OVX groups. CC inhibits serum biochemical markers in OVX-induced bone loss in mice.

The OVX group had reduced total femur BMD compared with the SH group, whereas CC treatment restored the BMD loss induced by OVX (Figure 6(a)). To investigate the effect of CC on OVX-induced bone morphometry, we analyzed trabecular architectural parameters in the distal femur using µCT analysis. In the two- and three-dimensional images, the deterioration of trabecular bone significantly increased in the OVX group compared with that in the SH group, whereas the CC-treated group showed markedly reduced OVX-induced deterioration in trabecular bone microarchitecture (Figure 6(b)). In the OVX group, the BV/TV, Tb.Th, and Tb.N were markedly lower but the Th.Sp was dramatically higher than the corresponding parameters in the SH group. On the other hand, BV/TV, Tb.th, and Tb.Sp increased in the CC-treated group compared with those in the OVX group. However, no significant effect on Tb.N was observed among the groups (Figure 6(c)).

**Figure 7:**
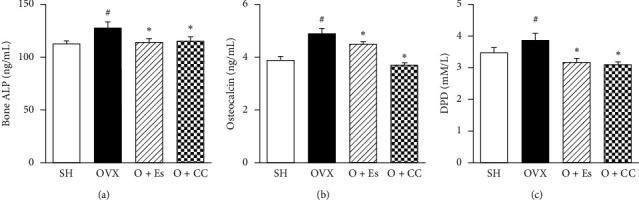
The effect of *Chrysanthemum coronarium* on bone turnover in mice. Serum levels of (a) bone-specific alkaline phosphatase (ALP), (b) osteocalcin, and (c) deoxypyridinoline (DPD) were measured. SH (sham), sham mice; OVX (ovariectomy), OVX mice; *O* + Es (estrogen), OVX + Es treatment mice; *O* + CC (*Chrysanthemum coronarium*); and OVX + CC treatment mice. Data are represented as means ± SEM. ^#^*p* < 0.05 compared with SH groups and ^*∗*^*p* < 0.05 compared with OVX groups.

To investigate the effect of CC on bone turnover biomarkers, we measured serum bone formation markers, including bone ALP and osteocalcin, as well as the bone resorption marker, deoxypyridinoline (DPD), using ELISA. The OVX group showed increased bone ALP, osteocalcin, and DPD. However, the CC extract was shown to significantly inhibit the level of bone ALP, osteocalcin, and DPD in the OVX-induced bone loss mouse model (Figure 7).

## 4. Discussion

With the increase in age and life expectancy, age-related diseases, such as osteoporosis, have become a serious public health problem. Osteoporosis is characterized by the progressive loss of bone mass and bone density, resulting in reduced bone strength and increased bone fractures [22]. Bone remodeling is essential to maintain a balance in bone formation by osteoblasts and bone resorption by osteoclasts. However, excessive bone resorption can lead to osteoporosis [23]. Recent treatment and prevention strategies of osteoporosis involve the use of antiresorptive agents (bisphosphonates and selective estrogen receptor modulators) and anabolic agents (parathyroid hormone) [24]. Although these agents are effective, their long-term use is associated with several side effects such as breast cancer, ovarian cancer, and cardiovascular diseases [25]. Therefore, there is a need to search for effective alternative approaches with less undesirable side effects to prevent and treat osteoporosis. Our study demonstrated that CC inhibits osteoclast differentiation, stimulates osteoblast differentiation, and protects against OVX-induced bone loss. In this study, Es was selected as a positive control because it has been demonstrated to exert beneficial effects on osteoporosis in OVX mice.

Osteoblasts, the bone-forming cells, are controlled by several factors, such as proliferation, collagen synthesis, mineralization, and deposition of the extracellular matrix of the bone [26, 27]. ALP is responsible for mineralization by initiating and/or promoting bone formation, and its level increases during osteoblastogenesis. ALP is known as a parameter of bone metabolism. Mineralization of osteoblasts leads to calcium deposition. Calcium-rich deposits in tissues were previously assessed using alizarin red S [28, 29]. In this study, the treatment of MC3T3-E1 cells with CC extract significantly stimulated osteoblast differentiation by enhancing ALP activity and calcium deposition (Figure 2).

Osteoblast differentiation is regulated by several marker genes, including ALP, Runx2, and osteocalcin in osteoblast cells [20]. ALP induces osteoblast differentiation, leading to an active formation of new bones. Runx2 is an essential transcription factor for proper osteoblast differentiation; its expression is upregulated in immature osteoblast cells. Additionally, osteocalcin is secreted from osteoblasts and plays an important role in the regulation of bone metabolism [30]. Park et al. reported that osteoclastogenesis is related to the high expression of these bone metabolic markers [30]. Our results showed that CC treatment increased ALP gene expression. Runx2 is a central transcription factor and is expressed in preosteoblasts, and its expression increases in immature osteoblast cells [31]. Runx2-deficient mice reportedly show complete inhibition of osteoblast differentiation and bone formation [32]. However, in our study, CC did not stimulate Runx2 expression in either mature osteoblasts or immature osteoblasts. Thus, further studies are needed to investigate the effects of CC on the differentiation of osteoblast precursors into immature and mature osteoblasts.

RANKL, produced by osteoblasts and other stromal cells, binds RANK and promotes osteoclastogenesis. OPG, which binds RANKL, is expressed by osteoblasts and prevents RANK signaling, acting as a decoy receptor and inhibiting osteoclast activation [33, 34]. Thus, the OPG/RANKL ratio is critical for the coupling of bone resorption to bone remodeling. In this study, we showed that CC increased the mRNA OPG/RANKL ratio in MC3T3-E1 cells (Figure 3). Therefore, these data suggest that CC exhibits beneficial effects on the bone remodeling process.

Osteoclasts are bone-resorptive cells that exist as osteoclast precursors that differentiate into activated multinucleated mature osteoclasts by RANKL and monocyte/macrophage colony-stimulating factor [35, 36]. Osteoclast differentiation can be assessed by a series of markers, including TRAP, cathepsin K, Dc-stamp, and CTR [19]. We found that CC inhibited both TRAP activity and the staining of TRAP-positive cells but did not cause any cytotoxicity (Figure 4). CC significantly decreased the expression of osteoclast-related genes, such as TRAP, cathepsin K, Dc-stamp, and CTR (Figure 5). These results suggest that CC suppresses osteoclast differentiation by downregulating osteoclastic markers.

OVX mice with estrogen deficiency are considered an acceptable model to investigate osteoporosis in postmenopausal women. OVX induces obesity and cardiovascular disease development [37]. In this study, the OVX group showed increased body weight gain, which was decreased by CC (Table 2). This result suggests that CC prevents obesity in OVX mice. Furthermore, OVX mice showed increased uterine atrophy, which was alleviated by CC, suggesting that CC may play a weak estrogenic role.

OVX mice also show reduced bone mass and deterioration of the trabecular structure, similar to the observation in humans [38, 39]. BMD is the measurement of bone strength and bone quality and is an important factor for the assessment of osteoporosis risk. Furthermore, microarchitecture assessments are necessary to evaluate the true impact of a treatment on the quality of trabecular bone. Both BMD and trabecular bone microarchitecture may improve bone strength [40]. In our study, OVX mice showed a marked decrease in BMD and trabecular bone structures. However, CC significantly restored the OVX-induced bone loss and trabecular bone architectures *in vivo* (Figure 6). These findings demonstrate that CC prevents bone loss and improves the deterioration of the trabecular bone structure.

Rapid bone loss enhances bone remodeling as determined by the increased levels of biochemical markers of bone turnover and biomarkers of osteoblastic activity, such as ALP and osteocalcin, and biomarkers of bone resorption, such as DPD [41–43]. In this study, OVX mice showed significantly increased levels of serum ALP, osteocalcin, and DPD, indicating increased bone turnover. However, CC treatment significantly decreased this increase in serum levels of ALP, osteocalcin, and DPD in the OVX group (Figure 7). These findings suggest that CC prevents bone loss by decreasing bone turnover.

Takenaka et al. reported that CC extracts contain caffeic acid and chlorogenic [15]. Compounds involved in CC extracts––caffeic acid and chlorogenic acid––prevent ovariectomy-induced bone loss [35, 44]. Several studies have demonstrated that rutin has several biological effects, including antioxidative and anti-inflammatory effects [44, 45]. Moreover, Horcajada-Molteni et al. reported that rutin also inhibits ovariectomy-induced bone loss [46]. Furthermore, caffeoylquinic acid has antioxidative and anti-inflammatory effects, and the caffeoylquinic acid-rich fraction of *Periploca forrestii* exhibits anti-inflammatory and antiarthritic activities [47–49]. In addition, some recent studies have demonstrated that an increase in oxidative stress assists bone resorption by promoting osteoclast differentiation [50]. Still, further studies will be needed to investigate these extracts and compounds antioxidant activities and the possible consequent antiosteoporotic effects in depth.

## 5. Conclusions

In conclusion, our results demonstrated that CC treatment prevents OVX-induced bone loss and deterioration of the trabecular microarchitecture. The mechanism of action of CC is mediated by the stimulation of osteoblast formation and the reduction of osteoclast resorption. Thus, CC extract may be a good candidate for preventing osteoporotic bone loss.

## Figures and Tables

**Table 1:** Sequences of target gene-specific primers used in RT-qPCR.

Target gene		Sequence
*Cathepsin K*	Sense Antisense	5′-CAG TAG CCA CGC TTC CTA TCC -3′ 5′-ACT GGG TGT CCA GCA TTT CC-3′

*ctr*	Sense Antisense	5′-GGAAAAGAAGTGCCCGCTGA-3′ 5′-TCTCCACTGA AAGCCAGCAG-3′

*trap*	Sense Antisense	5′- GCA GCC AAG GAG GAC TAC-3′ 5′-CCC ACT CAG CAC ATA GCC-3′

*DC-stamp*	Sense Antisense	5′- TAT CTG CTG TAT CGG CTC A-3′ 5′-AGA ATA ATA CTG AGA GGA ACC CA-3′

*alp*	Sense Antisense	5′- CGGGACTGGTACTCGGATAA -3′ 5′-TGAGATCCAGGCCATCTAGC-3′

*runx2*	Sense Antisense	5′- CGGCCCTCCCTGAACTCT -3′ 5′-TGCCTGCCTGGG ATCTGTA-3′

*opg*	Sense Antisense	5′-GTTCTTGCACAGCTTCACCA-3′ 5′-AAACAGCCCAGTGACCATTC-3′

*rankl*	Sense Antisense	5′-CCCTGAAAGGCTTGTTTC-3′ 5′-CCATGAAAACGCAGATTTG-3′

*gapdh*	Sense Antisense	5′-AAA TGG TGA AGC TCG CTC TG-3′ 5′-TGA AGG GGT CGT TGA TGG-3′

*ctr*, calcitonin receptor; *trap*, tartrate-resistant acid phosphatase; *DC-stamp*, dendritic cell-specific transmembrane protein; *alp*, alkaline phosphatase; *runx-2*, runt-related transcription factor-2; *opg*, osteoprotegerin; *rankl*, receptor activator nuclear factor-*κ*B ligand; *gapdh*, glyceraldehyde 3-phosphate dehydrogenase.

**Table 2:** Effects of *Chrysanthemum coronarium* on body, fat, liver, and uterine weights and food intake of OVX mice.

		SH	OVX	O + Es	O + CC
Body weight (g)	Initial	28.4 ± 0.8	29.2 ± 0.5	28.7 ± 1.2	29.6 ± 0.9
	Final	39.3 ± 2.4	57.2 ± 1.6^###^	35.2 ± 0.8^*∗∗∗*^	50.1 ± 2.4^*∗*^
Food intake (g/day)		8.0 ± 0.6	8.1 ± 0.5	7.4 ± 0.4^*∗*^	8.3 ± 0.2
FER		5.0 ± 3.9	7.1 ± 3.5	5.9 ± 3.0	6.0 ± 2.4
Fat (g)		0.36 ± 0.12	0.71 ± 0.02^###^	0.18 ± 0.02^*∗∗∗*^	0.59 ± 0.03^*∗*^
Uterus weight (g)		0.45 ± 0.03	0.05 ± 0.007^###^	0.36 ± 0.003^*∗∗∗*^	0.078 ± 0.002^*∗*^

SH (sham), sham mice; OVX (ovariectomy), OVX mice; O + Es (estrogen), OVX + Es treatment mice; O + CC (*Chrysanthemum coronarium*), OVX + CC treatment mice. FER indicates food efficacy rate (body weight gain/g feed). Data are represented as means ± SEM, *n* = 8.^###^*p* < 0.001 compared with SH groups, ^*∗*^*p* < 0.05 and ^*∗∗∗*^*p* < 0.001 compared with OVX groups.

## Data Availability

The data used to support the findings of this study are available from the corresponding author upon request.

## Abbreviations

AA: Ascorbic acid
ALP: Alkaline phosphatase
*β*-GP: *β*-Glycerophosphate
BMD: Bone mass density
BV/TV: Bone volume to tissue volume ratio
CC: *Chrysanthemum coronarium L.*
CTR: Calcitonin receptor
DC-stamp: Dendritic cell-specific transmembrane protein
DPD: Deoxypyridinoline
GAPDH: Glyceraldehyde 3-phosphate dehydrogenase
OPG: Osteoprotegerin
OVX: Ovariectomy
RANKL: Receptor activator nuclear-factor *κ*B ligand
Runx-2: Runt-related transcription factor-2
Tb. Th: Trabecular thickness
Tb. N: Trabecular number
Tb. Sp: Trabecular separation
TRAP: Tartrate-resistant acid phosphatase.
